# Effects of Kinesiology Tape on Non-linear Center of Mass Dispersion During the Y Balance Test

**DOI:** 10.3389/fphys.2018.01527

**Published:** 2018-10-31

**Authors:** Pauline Andreo, Kinda Khalaf, Lainey Heale, Herbert F. Jelinek, Luke Donnan

**Affiliations:** ^1^Department of Pure and Applied Sciences, University of Poitiers, Poitiers, France; ^2^Department of Biomedical Engineering, Khalifa University of Science and Technology, Abu Dhabi, United Arab Emirates; ^3^School of Community Health, Charles Sturt University, Albury, NSW, Australia

**Keywords:** postural stability, center of mass, balance test, fractal dimension, ankle taping, complex movement

## Abstract

Static taping of the ankle or knee joint is a common method of reducing risk of injury by providing mechanical stability. An alternative taping technique employs kinesiology tape, which has the additional benefit of improving functionality by stimulating proprioception. There is substantial disagreement whether kinesiology tape shows significant differences in proprioception and postural stability as compared to rigid/static tape when applied at the lower limb. The current study investigated the effects of kinesiology tape and static tape during a Y Balance Test on center of mass as an indicator for postural stability. Forty-one individuals, free of injury, performed the Y Balance Test under the three conditions; no tape, kinesiology tape, and static tape applied at the lower limb to the quadriceps, triceps surae and ankle joint. All participants completed the Y Balance Test to determine whether any significant differences could be observed using center of mass movement as a surrogate measure for balance and proprioception. The Minkowski-Bouligand and box-counting fractal dimension analyses were used as measures of the dynamic changes in the center of mass whilst undertaking the Y Balance Test. Statistical analyses included the Kruskal Wallis test to allow for non-normally distributed data and a Bonferroni corrected pairwise *T*-test as a *post hoc* test to ascertain pairwise differences between the three taping conditions. Significance was set at 0.05. The fractal analyses of the dynamic changes in center of mass showed significant differences between the control and both the static tape and kinesiology tape groups (*p* = 0.021 and 0.009, respectively). The current study developed a novel measure of dynamic changes in the center of mass during a set movement that indicated real-time processing effects during a balance task associated with the type of taping used to enhance postural stability.

## Introduction

### Proprioception and balance

Proprioception is a sensory modality important in monitoring body position in space, balance and movement (Lephart et al., 1997). The somatosensory, vestibular, and visual systems are all involved in proprioception to retain balance and posture and to enable dynamic movements (Woollacott and Shumway-Cook, 2002). The receptors in the skin, muscles, ligaments, and tendons, as well as vestibular and visual information associated with proprioception, provide input to the central nervous system regarding body position. Taping of the lower limb has been used to improve stability using static tape or to enhance proprioception by applying kinesiology tape. The effectiveness of either taping method is best analyzed by measuring the degree of postural control during a balance test. Current measures are based on Euclidean geometry such as area covered by postural sway. Non-Euclidean or complexity measures such as fractal analysis are better suited to determine extent of postural sway and taping effectiveness.

### Assessment of body balance

Two tests are preferentially used to assess dynamic balance: the Star Excursion Balance Test (SEBT), and the Y Balance Test (YBT). These tests can be used to evaluate physical performance, investigate dynamic postural control, identify athletes at greater risk for injury, and provide quantitative data during rehabilitation. Both tests require reaching of the non-stance leg in set directions from a central standing position. The YBT uses three (anterior, posteromedial, and posterolateral) of the eight SEBT directions. No significant differences in terms of reliability and information gained from the two tests have been reported (Bell et al., 2011; Coughlan et al., 2012). The Balance Error Scoring System (BESS) and the Biodex Balance System are two additional measures of balance (Arnold and Schmitz, 1998). However, these tests do not measure center of mass (CoM) changes during dynamic movement, but rather assess the effect of a movement upon completion. Therefore, a more direct measure of proprioception and balance as key factors influencing the CoM during movement that assesses postural stability is required (Fritschi et al., 2014).

### Taping the lower limb

Postural stability is important during all types of tasks as part of activities of daily living and sport. The use of tape on the lower limb is often applied to improve dynamic postural control and has been investigated by several researchers (Briem et al., 2011; Nakajima and Baldridge, 2013; Hosp et al., 2015). Static or non-elastic tape (ST) is commonly used to limit the range of motion in a desired direction. This type of tape has been shown to be effective in reducing the prevalence of lower limb injuries (Karlsson et al., 1993; Wilkerson, 2002; Lardenoye et al., 2012; Jackson et al., 2016). However the mechanism responsible for improving postural control has not been clearly elucidated, and may be a function of the reduction in joint flexibility by the rigid tape or due to enhanced proprioceptive input from skin sensory receptors as suggested by the distributors of the kinesiology tape (KT; Corporation, 2017). For three decades KT has been adopted by athletes world-wide, but no clinical study has yet unequivocally shown that KT taping on the lower limb has better outcomes compared to ST for dynamic postural stability. One study reported that application of KT at the knee did not improve knee proprioception in healthy women, yet enhanced proprioception in women with poor proprioceptive ability (Hosp et al., 2015). A further study using the Balance Error Scoring System (BESS) investigated the effects of KT on balance deficits associated with chronic ankle instability. This study concluded that KT improved balance after it had been applied for 48 h, when compared with the pre-test and with the control group results (Jackson et al., 2016). Another study compared taping the ankles with kinesiology tape to rigid tape in male athletes undertaking the SEBT and found no significant effect on muscle activity (Briem et al., 2011). Although rigid tape and not kinesiology tape increased muscle activity when the ankles were taped, no correlation between muscle activation and the influence of either tape on postural stability could be determined. Finally, Nakajima et al. used the SEBT to analyse the effect of KT on dynamic postural control. They concluded that KT had an effect in females only in the posterior-medial and medial directions of the SEBT test (Nakajima and Baldridge, 2013).

### Fractal analysis as a dynamic measure

Measures of spatial dispersion of the CoM to investigate the effects of taping on movement have not been investigated using non-linear methods, although they provide means for dynamic assessment of CoM changes during the YBT or other balance tests. Current center of pressure (CoP) or center of mass analyses are based on position time series but also include spatial measures such as sway path length, the area covered by CoP/CoM dispersion, as well as range, maximal sway trajectory and peak velocity (Yamamoto et al., 2015). Thus, CoM can be analyzed as a time series associated with changes in a set direction of movement or as a 2D trajectory or spatial dispersion as a function of postural control during the YBT. Better postural stability is associated with greater variation and dispersion in the CoM trajectory leading to a more complex geometric pattern. Postural stability which is associated with variability in the CoM trajectory and observed during movement is a non-linear phenomenon and is not well described by Euclidean geometry. Fractal geometry may be useful for measuring the CoM trajectory associated with YBT and any changes due to taping (Huang et al., 2013; Gilfriche et al., 2018). The fractal dimension (FD) is a descriptive parameter, which can provide an index of the complexity of a non-linear pattern such as the CoM trajectory. Several fractal analyses methods have been applied to investigate non-linear spatial distributions, among them the box-counting and Minkowski-Bouligand methods are commonly used. The fractal dimension is a useful parameter for classifying complex patterns (Jelinek and Fernandez, 1998) and several studies have described changes in postural stability and balance including the ability of postural adjustments in patients with Prader-Willi Syndrome (Błaszczyk and Klonowski, 2001; Cimolin et al., 2011). The Prader-Willi Syndrome study used the box-counting method to investigate traces of CoP trajectories. The authors demonstrated that the CoP trajectories in Prader-Willi Syndrome were characterized by higher values of FD when compared to a control group. A study investigating ankle sprain injuries, however, indicated a reduction in fractal dimension (Doherty et al., 2015). These two studies suggest that a fractal dimension value that is too small or too large indicates compromised proprioceptive function.

The current study investigated the spatial dispersion of CoM associated with the YBT in a healthy cohort of young adults and used Minkowski-Bouligand and box-counting methods to determine the fractal dimension to evaluate the effect of static vs. kinesiology tape applied to the lower limb compared to no tape.

## Methods

### Participants

A total of 41 participants were recruited at Charles Sturt University for the study. The participants completed a questionnaire reporting injuries for the last 5 years. Age, gender, sporting activity, frequency and amount, individual height, and weight for all participants were noted. The Human Research Ethics Committee of Charles Sturt University approved the protocol (protocol number H16113). All participants provided written consent to take part in the research following receipt of an information package.

### Taping procedures

**Figure 1:**
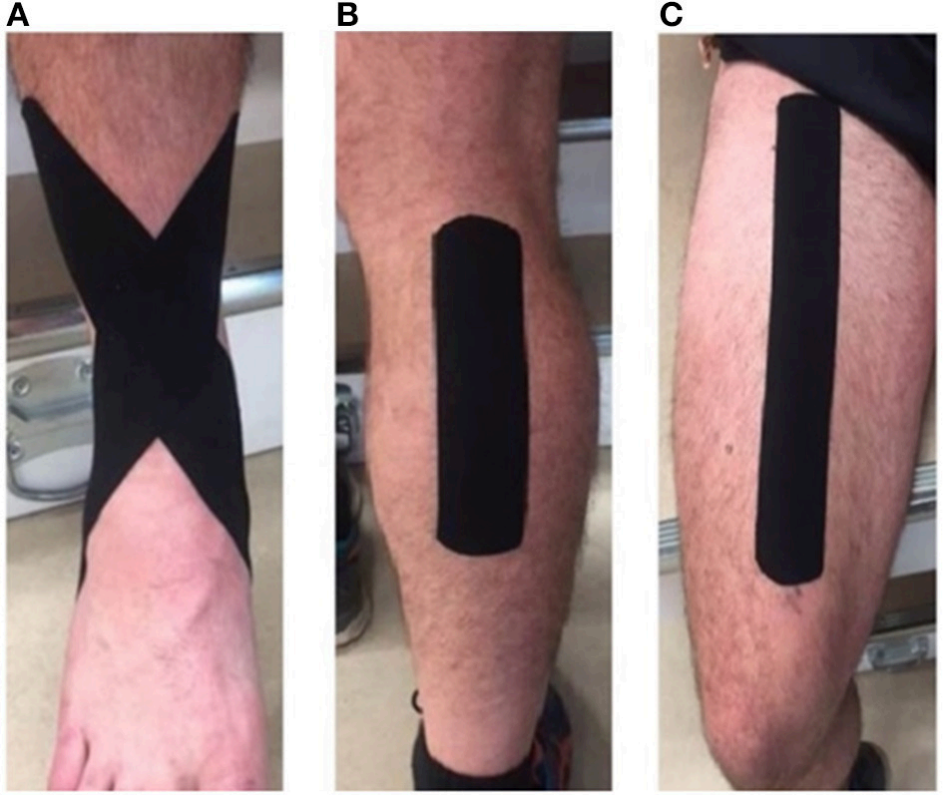
Taping procedures at **(A)** the ankle, **(B)** the calf, and **(C)** the quadriceps.

All participants were tested on two separate occasions, using kinesiology tape (Kinesio Tex®) on one occasion, and rigid tape on the other. The order of tape allocation was randomized. Both occasions included a no Tape trial. A single strip of tape was applied without tension to the muscle belly of rectus femoris and the medial head of gastrocnemius while the muscle was under stretch. An anti-inversion ankle strapping was also applied using a single strip of tape without tension starting from the medial lower leg, passing inferiorly over the lateral malleolus (lateral part of the ankle), under the arch of the foot, passing superiorly over the medial malleolus, and finishing at the lateral aspect of the lower leg (Van Den Dries et al., 2013). As the YBT utilizes sagittal plane motion at the knee, and a combination of sagittal and frontal plane motion at the ankle, three strips were applied to potentially influence the three fundamental movements requiring control throughout the task. All strapping was applied following procedures outlined in the guide produced by one KT manufacturer (Capobianco and Van Den Dries, 2013), and applied to the dominant leg (Figure 1).

### Y balance test

**Figure 2:**
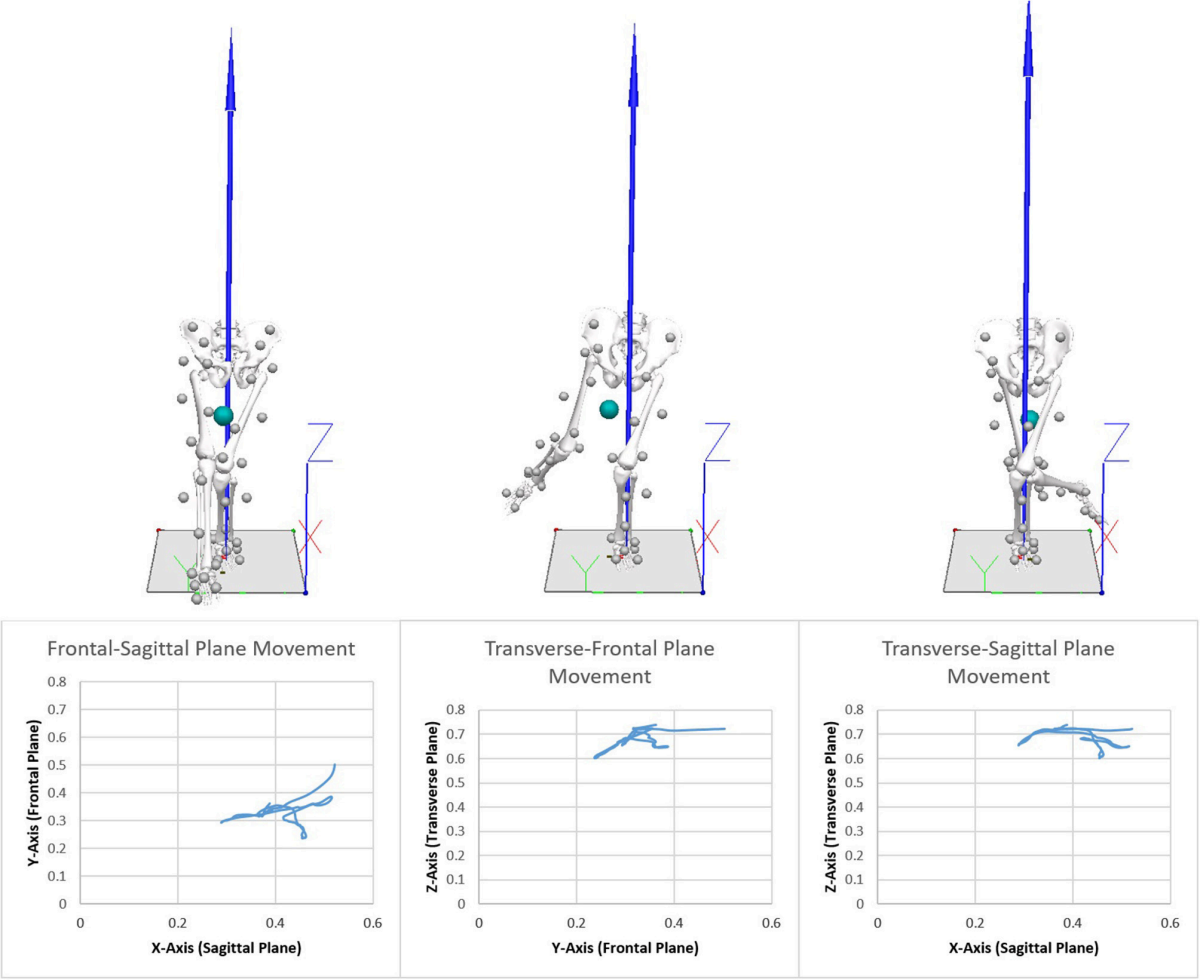
An illustrative example of a Vicon 3D frame.

As a method of assessing dynamic postural stability, the Y Balance test (YBT) was used to investigate the effect of taping. The goal of the YBT is to reach as far as possible with one leg in three directions while maintaining balance with the dominant leg. The participant stands at a central point and extends the non-dominant leg anteriorly, posterolaterally, and posteromedially (Figure 2). Each participant performed one practice trial to become familiar with the test, before performing five trials with no tape, KT and ST tapes, respectively. On the first testing occasion, the participant performed the YBT untapped and with one of the taping variables (KT or ST). A minimum of 3 days later, the participant performed the YBT again, both untapped and then with either the ST or KT (the tape not used on the first occasion). The minimum 3 days break between testing was used as a washout period to ensure that the results would reflect the taping intervention, not the carry over effects from previous testing (Chow and Liu, 2004). In addition, the 3-day interval also showed whether there was a difference in response to the YBT when no tape was applied. An eight-camera 3D motion capture system (100 Hz, Vicon, Oxford Metrics, UK) was used to obtain kinematic data based on 36 reflective markers applied to the pelvis and lower limb, while an AMTI force plate (1000Hz, AMTI, USA) collected kinetic data (Figure 2). Processes specific to Visual3D (Version 6, C-Motion, Germantown, MD) were used to calculate the mass of each segment, which allowed identification of the relative position of the center of mass. A fourth-order Butterworth low-pass filter was used to filter kinematic (18 Hz) and kinetic (50 Hz) data prior to export for statistical analysis. Of the 890 individual trials, the mean duration for the sequence of three reaches was 880.6 (± 314.6) frames (8.81 s). All trials were subsequently normalized to 1,001 frames to allow individual trials to be directly compared. In Figure 2, the vertical arrow represents the ground reaction force vector, the circle depicts the relative center of mass. The bottom row represents the CoM dispersion. While movement in the z-axis affects superior and inferior movement of the CoM, movement in the x and y-axis shifts CoM outside the base of support, which requires somatosensory and neuromuscular adaptation to maintain a balanced state. For this reason, the current analysis focused on the CoM dispersion associated with postural adjustment in a 2-dimensional representation in the x and y axis (anterior/posterior and medial/lateral dispersion). The non-linear spatial dispersion characteristics of CoM were determined using fractal analysis and more common linear features including total sway area and maximum/minimum trajectory.

### Fractal analysis

Traditional common features extracted from the spatial dispersion of the center of mass included total path length, maximum and minimum trajectory in each direction and total area (Yamamoto et al., 2015). Fractal analysis was performed on the 2D representation of the CoM trajectory during the YBT from initiation of the reach attempts in the anterior, posterior lateral and posterior medial direction and to return to the central position. The recorded CoM dispersion was then pre-processed by filtering any noise and analyzed.

#### Minkowski- bouligand method

**Figure 3:**
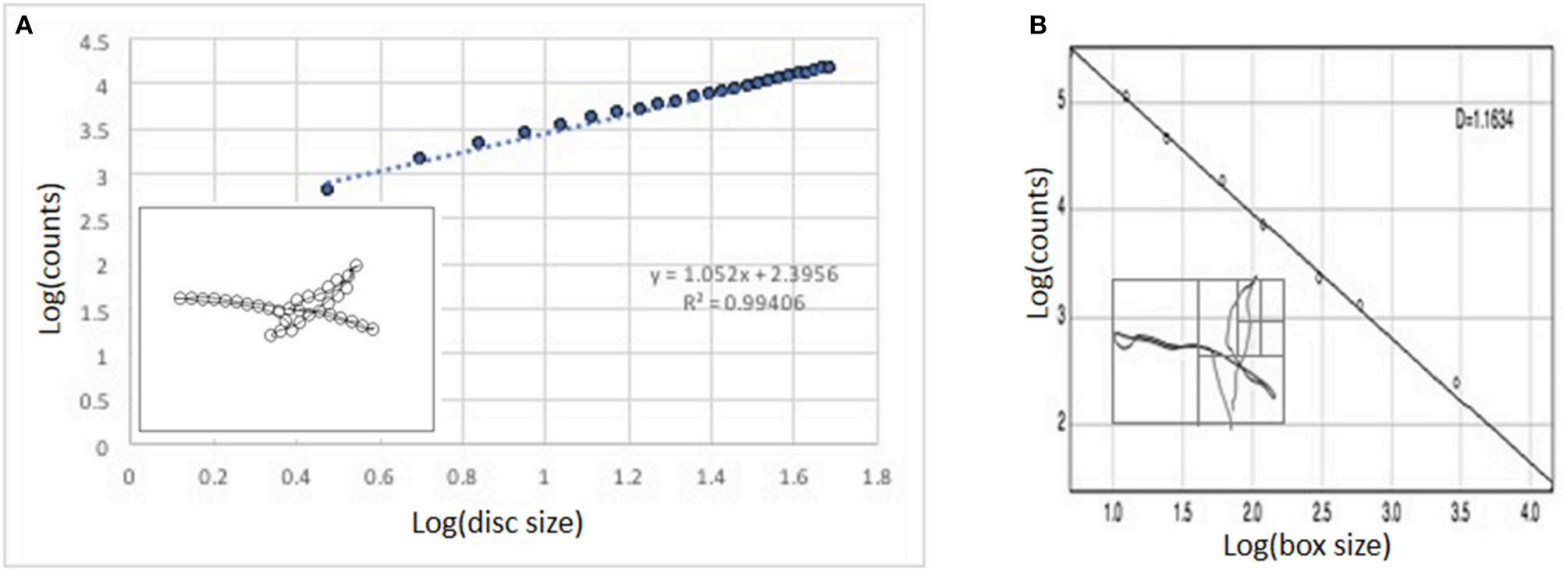
**(A)** Log-log plot of Minowski dimension analysis. **(B)** Log-log graph of Box counting dimension analysis.

A computer-based approach to measure the fractal dimension was developed by Tom Smith and his colleagues from existing mathematical concepts and implemented applying an in-house Minkowski-Bouligand dimension macro within NIH Image software (Smith and Behar, 1994; Smith et al., 1996; Jelinek and Fernandez, 1998). The Minkowski-Bouligand dimension is determined by replacing each pixel of a line representing the changes in the CoM associated with the YBT with an array of pixels or circles whose diameter increased with each pass (Figure 3). The double logarithm of the scale (circle diameter) and length of the line associated with the changes in CoM during the YBT result in a linear relationship (regression line) if the image is fractal over several generations of scales. The slope (S) of the regression line is used to calculate the fractal dimension (Fernández and Jelinek, 2001; Jelinek et al., 2005). The fractal dimension is calculated using the equation: Df_MB_ = 2–S.

#### Box-counting method

The box-counting dimension was determined using the FracLac box-counting algorithm in ImageJ (Karperien and Jelinek, 2015). The box-counting method divides the space which contains the image into equal sized boxes of progressively smaller sides. Then, the boxes that include part of the image of interest are counted (Figure 3) (Fernández and Jelinek, 2001). The double logarithm of scale vs. the number of filled boxes is an approximate straight line whose gradient is Df_BC_.

### Statistical analysis

The Shapiro-Wilk Test was used to check for normality as recommended for sample size below 50 and based on regression and correlation, whereas the Kolmogorov-Smirnov test is based on the empirical distribution function and only considers the largest discrepancy between observed and hypothesized distributions (Yap and Sim, 2011). The results showed a normal distribution. A repeated measures student *t*-test was performed to investigate whether significant differences existed between the first trial and the second trial performed without tape 3 days later. Data including fractal dimension, reach distance, and demographic characteristics of the participants were analyzed using a general linear mixed model (Cnaan et al., 1997). Linear mixed-effect models typically combine the components of fixed effects, random effects, and repeated measurements in a single unified approach which was appropriate for the study (Grajeda et al., 2016). The model was followed by a Bonferroni corrected pairwise *t*-test as a *post hoc* test to ascertain pairwise differences between the three conditions (no tape, KT, rigid tape). Spearman's correlation was determined to investigate whether the fractal dimension was correlated with maximum reach. Descriptive statistics were calculated for male and female groups. Significance was set at *p* < 0.05.

## Results

Forty-one participants attended the clinic to assess the effect of taping on balance. Of these, 21 were males and 20 were females. Table 1 represents the mean and standard deviation of age, height, and weight for each gender.

**Table 1:** Characteristics of participants for gender.

**Gender**	**Age (years)**	**Height (m)**	**Weight (kg)**	**BMI (kg/m^2^)**
Male	22.91 ± 3.61	1.81 ± 0.07	84.74 ± 10.53	25.67 ± 1.77
Female	21.05 ± 1.82	1.66 ± 0.06	66.57 ± 7.07	24.37 ± 3.35

*Mean ± standard deviation*.

Several variables can affect postural stability including being overweight or obese (Hue et al., 2007). Therefore, body mass index (BMI) was included in the analysis and normal range set at between 20 and 25 kg/m^2^. BMI was within the normal range for both genders with females having a slightly lower BMI but not significantly different. No significant difference was noted for age between the genders. The participants also recorded their activity profiles. This indicated that the majority of participants undertook on average 5 h of activity per week.

The change in the CoM dispersion for the no tape condition did not differ between males and females, and therefore male and female data were combined (Tables 2–4).

**Table 2:** Traditional linear measures of spatial patterns associated with CoM dispersion.

**Feature**	**NoTape**	**Static tape**	**Kinesiology tape**
**AVERAGE TRAJECTORY**			
Anterior	63.1 (± 17.3)	65.6 (± 18.9)	66.0 (± 20.7)
Posterior-medial	87.0 (± 13.4)	86.2 (± 16.8)	86.6 (± 13.3)
Posterior-lateral	70.1 (± 14.8)	71.1 (± 19.5)	70.7 (± 19.5)
**MAXIMUM TRAJECTORY (FOR ALL PARTICIPANTS)**			
Anterior	157.1	150.0	160.5
Posterior-medial	115.0	122.7	115.8
Posterior-lateral	152.0	150.9	147.9
**MINIMUM TRAJECTORY (FOR ALL PARTICIPANTS)**			
Anterior	36.1	26.9	31.4
Posterior-medial	43.7	47.3	53.8
Posterior-lateral	37.6	39.4	38.6

*Mean ± standard deviation. All data in centimeters*.

**Table 3:** Effects of taping measured with the Minkowski-Bouligand method.

	**Control**	**KT**	**ST**
Group	0.957 ± 0.005	0.974 ± 0.007	0.976 ± 0.007^*^
Males	0.957 ± 0.007	0.979 ± 0.010	0.976 ± 0.010
Females	0.957 ± 0.009	0.969 ± 0.009	0.976 ± 0.009

Mean ± standard error;

* *significant finding (p ≤ 0.05)*.

**Table 4:** Effect of taping measured by the box-counting method.

	**Control**	**KT**	**ST**
Group	1.173 ± 0.006	1.189 ± 0.008	1.197 ± 0.008^*^
Males	1.167 ± 0.008	1.186 ± 0.010	1.194 ± 0.010
Females	1.179 ± 0.010	1.192 ± 0.010	1.20 ± 0.010

Mean ± standard error;

* *significant finding (p ≤ 0.05)*.

Common features investigated to determine postural stability or sway were analyzed with respect to taping condition and YBT. The results are shown in Table 2. No significant effect of taping was observed for total path length, maximum, and minimum trajectory in each direction and total area. Supplementary File 1 offers a video of a single participant completing the YBT in the no tape condition first and followed by the static tape condition. It should be noted that while no significant effect was observed for common measures of postural stability and sway, an increase in corrective movements can be observed at the femur of the support leg in the static tape condition compared to the no tape condition. Results of the fractal analysis also indicated a significant effect of taping.

A linear mixed effects model was applied to consider the three experimental interventions of no tape, KT and ST tape as well as gender. The *t*-test revealed no significant difference between the first and third day trials. As such, the data was averaged and the effects of KT and ST were compared against this average control condition data. All data were normally distributed (Shapiro-Wilk Test >0.05). Table 3 shows the results for the Minkowski-Bouligand dimension analysis.

The box-counting results are shown in Table 4.

Fractal dimension results close to 1 may reflect a transition phase between Euclidean and fractal forms or are a function of the smoothness of the Minkowski-Bouligand dimension (dilation method) that is more sensitive to linear features.

Significant differences were found when the fixed effects were the Minkowski-Bouligand (Df_MB_) or box-counting (Df_BC_) method. The model showed a significant difference for KT and ST groups as compared to control with the Minkowski-Bouligand method (*p* = 0.0216 and *p* = 0.0098 respectively), and for ST with the box-counting method (*p* = 0.0048). The same model was applied for males only and females only. Analysing effects of taping within gender showed that both Df_MB_ and Df_BC_ were significant. A difference was found for KT for males according to the Df_MB_ method (*p* = 0.0464) and for ST according to the box-counting method (*p* = 0.0249) but not for females. A *post hoc* comparison using Bonferroni correction indicated a significant result for no tape vs. rigid tape for both the Minkowski-Bouligand and box-counting methods (*p* = 0.052 and *p* = 0.0093) but no significant difference within gender. The fractal data and maximum reach data showed that a correlation existed for anterior reach and posteromedial reach when using ST taping (*r* = 0.65 and *r* = 0.55, respectively). When maximum reach points in the three directions of the YBT were connected to form a sway area description of maximum reach in the three directions, there was no correlation between the fractal dimension results and the triangle area and perimeter (*r* = 0.04 and *p* = 0.9).

## Discussion

The purpose of this study was to investigate the effects of static and kinesiology taping on postural stability during a dynamic movement control test using the Y balance test. Postural control tasks, such as the YBT, allow investigation of movement and balance around a central support base. A change in the CoM trajectory and resulting fractal dimension indicates a change in the postural sensori-motor strategies applied during the YBT. The box-counting and Minkowski-Bouligand methods were used here to investigate changes in the CoM during the YBT. The principal finding of our study was a significant increase in the fractal dimension for rigid tape vs. no tape, when results were corrected for multiple group comparison. However, both taping conditions led to an increase in the Minkowski-Bouligand (Df_MB_) and box-counting (Df_BC_) dimensions. Larger fractal dimension values of the CoM trajectory are associated with greater activity of the sensori-motor system in retaining balance during a dynamic movement task. In contrast, in previous studies applying KT did not improve knee or ankle proprioception in a group of healthy young adults, did not decrease or increase performance, and had no effects on muscle activation (Briem et al., 2011; Nakajima and Baldridge, 2013; Hosp et al., 2015. Similar findings were reported when analyzing postural sway using multiscale entropy analysis, where postural sway dynamics of healthy subjects were more complex than that of subjects with a history of falls (Costa et al., 2007). The current data combined with previous data indicates what may seem contradictory results but confirms a physiological adaptation process or adaptive stress response to moderate intermittent stress occurring (Mattson, 2008). Normal physiological phenomena occur within a set range, where activity outside this range is considered pathological. Our results indicate that taping increased complexity of CoM dispersion suggesting an improvement in postural stability. However extensive increases in CoM dispersion has been observed in patients with Prader-Willi Syndrome and in patients prone to falls, which is suggestive of pathology and reduced proprioceptive ability and a reduction in postural control (Costa et al., 2007; Cimolin et al., 2011).

The linear mixed effects model which was used in the current study considers the repeated design nature of the research (no tape, KT, and ST conditions) and gender, and showed a significant difference in males for Df_MB_ when KT was applied and for Df_BC_ when ST was applied. The results suggesting that the fractal analysis method had an effect on the findings. ST led to greater complexity and spread of the CoM trajectory across movement space. In instances where the dynamics of the CoM are within a smaller movement space/envelope, as may be the case with KT, the box-counting method may be more sensitive. Our findings suggest that KT tape may decrease extreme postural sway better to maintain balance due to stimulation of skin receptors and heightened proprioceptive information provided by KT application. This agrees with previous data where KT was shown to improve postural stability in females only in the posterior-medial and medial directions of the SEBT test (Nakajima and Baldridge, 2013). KT also demonstrated significant proprioceptive enhancement at the knee joint after uphill walking in healthy women with poor proprioceptive ability (Hosp et al., 2015). Our results now extend this to a group of healthy young adults. The difference in gender found in the current study may be due to the fact that males and females use different sensori-motor strategies for postural control during movement (Wikstrom et al., 2006; Smith et al., 2012).

The fractal dimension reflects the degree of complexity associated with changes in the CoM trajectory in the anterior-posterior and medial–lateral directions during the YBT. In general, a higher fractal dimension represents more complex movement patterns, possibly reflecting better dynamic control and postural stability (Costa et al., 2007). Conversely, a lower fractal dimension represents reduced dynamic control and less postural stability possibly due to compromised somatosensory feedback. However, fractal analyses results indicating a very much lower or higher fractal dimension are likely indicative of sensori-motor dysfunction and loss of postural stability. A previous study, investigating immediate post-injury movement strategies with SEBT and using the center of pressure to calculate the fractal dimension with a similar method to the dilation method (Katz, 1988) demonstrated a reduction in the fractal dimension for participants with a lateral ankle sprain. The authors interpreted this result as a reduced ability to perform the balance test (Doherty et al., 2015). Pain is known to restrict movement, especially when a maximum reach in any direction is required, and hence the fractal dimension is expected to decrease. Other studies in which control participants were age-matched with patients affected by Prader-Willi Syndrome computed the fractal dimension on the image of the CoP trajectory using the box-counting method and showed that patients with Prader-Willi syndrome were characterized by higher values in the fractal dimension and a poorer balance capacity when compared to the control group (Capodaglio et al., 2011; Cimolin et al., 2011). The higher fractal dimension was interpreted as the inability for these patients to modulate the sensori-motor systems involved in postural control, possibly related to compromised sensori-motor feedback at the spinal cord, brainstem or subcortical/cortical level(s) (Cimolin et al., 2011).

In our study, with a young adult cohort free of injury, the fractal dimension for KT and ST was higher than the control for both methods. This suggests that rigid or kinesiology tapes applied on healthy subjects may improve postural stability. Rigid tape led to slightly higher Df_MB_ and Df_BC_ that could be due to ST decreasing joint movement more than KT. A previous study which investigated the fractal structure of force plate signals suggested that the center of pressure was more useful and sensitive in the evaluation of the age-related decline of postural stability than the CoM (Błaszczyk and Klonowski, 2001). The current study utilized the CoM, and future studies need to investigate the difference between the CoP, which is more sensitive for vertical force distribution through the standing leg, and CoM, which is more sensitive to body sway and movement adjustment. In addition, to determine whether KT does in fact have a significant effect that is different from ST, a larger cohort study is being organized as well as different fractal analysis methods incorporated such as the mass-radius and caliper methods. A future study should include measures of nerve conduction velocity to control for sensory information processing and two-point discrimination assessment in the lower limb as a baseline measure of skin sensation sensitivity.

An association between fractal dimension and maximum leg extension was not found in the current study, suggesting that the CoM is not a function of maximum reach. Consequently, the improved postural stability may not be associated with a shorter reach. According to this result, the fractal dimension represents the dynamics of movement and is not a function of the maximum reach of any movement, which suggests that taping in general does not affect the extent of movement but rather postural stability.

The findings of this study indicate both KT and ST application contribute to increased complexity of CoM movement during a dynamic balance task, however it was only ST that achieved significance when compared to no tape. This is contrary to claims KT has the potential to facilitate changes in proprioception and postural stability. While the findings are of clinical interest, the use of fractal analysis to achieve these findings offers a novel, and potentially more appropriate method for future investigation of movement complexity.

## Author contributions

LD and HJ conceived of the study, interpreted the results and finalized the manuscript. KK contributed to interpretation of results and writing the manuscript. LH collected the data, analyzed the data, and contributed to the writing of the manuscript. PA undertook the fractal analysis and contributed to the interpretation of the results and writing of the manuscript.

### Conflict of interest statement

The authors declare that the research was conducted in the absence of any commercial or financial relationships that could be construed as a potential conflict of interest.
